# Stated product formulation preferences for HIV pre-exposure prophylaxis among women in the VOICE-D (MTN-003D) study

**DOI:** 10.7448/IAS.19.1.20875

**Published:** 2016-05-30

**Authors:** Ellen H Luecke, Helen Cheng, Kubashni Woeber, Teopista Nakyanzi, Imelda C Mudekunye-Mahaka, Ariane van der Straten, on behalf of the MTN-003D Study Team

**Affiliations:** 1 Women's Global Health Imperative (WGHI) RTI International, San Francisco, CA, USA; 2 South African Medical Research Council, HIV Prevention Research Unit, Durban, South Africa; 3 Makerere University – Johns Hopkins University Research Collaboration, Kampala, Uganda; 4 UZ-UCSF Collaborative Research Programme, Harare, Zimbabwe; 5 Center for AIDS Prevention Studies (CAPS), University of California, San Francisco, CA, USA

**Keywords:** vaginal microbicides, oral pre-exposure prophylaxis, Africa, women, HIV prevention, product delivery forms, preferences

## Abstract

**Introduction:**

The effectiveness of HIV pre-exposure prophylaxis (PrEP) requires consistent and correct product use, thus a deeper understanding of women's stated product formulation preferences, and the correlates of those preferences, can help guide future research. VOICE-D (MTN-003D), a qualitative ancillary study conducted after the VOICE trial, retrospectively explored participants’ tablet and gel use, as well as their preferences for other potential PrEP product formulations.

**Methods:**

We conducted an analysis of quantitative and qualitative data from VOICE-D participants. During in-depth interviews, women were presented with pictures and descriptions of eight potential PrEP product formulations, including the oral tablet and vaginal gel tested in VOICE, and asked to discuss which product formulations they would prefer to use and why. Seven of the original product formulations displayed were combined into preferred product formulation categories based on exploratory factor and latent class analyses. We examined demographic and behavioural correlates of these preferred product formulation categories. In-depth interviews with participants were conducted, coded, and analysed for themes related to product preference.

**Results:**

Of the 68 female participants who completed in-depth interviews (22 South Africa, 24 Zimbabwe, 22 Uganda), median age was 28 (range 21–41), 81% were HIV negative, and 49% were married or living with a partner. Four preferred product formulation categories were identified via exploratory factor analysis: 1) oral tablets; 2) vaginal gel; 3) injectable, implant, or vaginal ring; and 4) vaginal film or suppository. A majority of women (81%) expressed a preference for product formulations included in category 3. Characteristics significantly associated with each preferred product category differed. Attributes described by participants as being important in a preferred product formulation included duration of activity, ease of use, route of administration, clinic- versus self-administration, and degree of familiarity with product.

**Conclusions:**

While there was interest in a variety of potential PrEP product formulations, a majority of VOICE-D participants preferred long-acting methods. More research is needed to gain insight into end-users’ product formulation preference to inform messaging and market segmentation for different PrEP products and resources to invest in products that target populations are most interested in using.

Clinical Trial Number: NCT02358616

## Introduction

Despite tremendous progress in curbing the global epidemic, sub-Saharan Africa continues to experience the highest HIV incidence rates, particularly among young women [1]. Women are at increased biological and social vulnerability to HIV infection, but producing an HIV prevention product that women will use has been an ongoing challenge. Oral pre-exposure prophylaxis (PrEP) has been found to be effective among men and women in four trials [2–5], and oral tenofovir-based PrEP is now approved as a highly effective HIV prevention strategy when used consistently. Additionally, one proof of concept trial has reported modest protection with pericoital dosing of a vaginal gel [6]. However, two PrEP trials with daily dosing among female study populations [7,8] and one confirmatory trial of pericoital vaginal gel dosing [9] had very low product adherence and were unable to demonstrate effectiveness. Two large Phase III clinical trials have shown that a monthly vaginal ring delivering antiretrovirals (ARVs) can significantly reduce HIV infections in women [10,11]. Notably, both studies reported greater protection in women who had higher evidence of ring use, including those over age 21. Thus, the promise of PrEP for HIV prevention in young women depends upon their willingness and ability to correctly and consistently use the prevention technologies that are being developed for them.

Use of HIV prevention products is not driven by a diagnosis or relief of symptoms, and product use requires women to participate in preventive health behaviours. Product acceptability depends upon experiences, perceptions of product attributes, and use requirements [12]. These individual-level factors must also be considered within the context of their sexual relationships, their communities and culture, and in the context of clinical trial participation for product evaluation [12,13]. Further, women's diverse needs for HIV prevention, which change over the course of their lifetime, will be best met if multiple delivery forms with varying attributes and use requirements are available [12,14–18].

The VOICE-D (MTN-003D) qualitative ancillary study was conducted following the Phase IIB VOICE (MTN-003) trial, which studied the safety and effectiveness of two different HIV prevention approaches among women in Uganda, South Africa, and Zimbabwe: daily use of an ARV tablet (tenofovir or Truvada) or daily use of a vaginal gel (tenofovir gel). The VOICE-D sub-study was designed to retrospectively explore participants’ tablet and gel use, as well as their preference for other potential HIV PrEP products. In this article, we explored women's product preferences, the correlates of those preferences, and explanations for their selections.

## Methods

### VOICE-D study

VOICE-D was a two-stage qualitative ancillary study implemented following the VOICE trial, with the aim of better understanding women's actual and reported use of study products and sexual behaviour during the VOICE trial. The VOICE trial (MTN-003; ClinicalTrials.gov identifier: NCT00705679, detailed in [8]) was conducted from 2009 to 2012 among 5029 women at 15 sites in Uganda, South Africa, and Zimbabwe. For the VOICE-D study, former VOICE participants were recruited from among those who had provided permission to be recontacted and had plasma tenofovir (TFV) data.

This article includes data collected during the second stage of the VOICE-D study (November 2013–March 2014). The VOICE-D study stage 2 (detailed in [19]), enrolled former VOICE participants assigned to active study product arms. Enrollment was stratified by HIV-seroconversion status, tablet or gel assignment, and plasma TFV pharmacokinetic (PK) level during VOICE (low: no plasma TFV detected at any visit, inconsistent: plasma TFV detected at 1-74% of visits, and high: plasma TFV detected at 75-100% of visits). Women were systematically selected to participate in in-depth interviews (IDIs) (detailed in [19]). Those in IDIs were prompted towards the end of the interview to choose what type of product formulation(s) they would take, if any, for HIV prevention from a list of eight options and were then asked to explain their selection(s).

### Procedures

IDIs were conducted by trained female research staff that were not a part of the VOICE trial at a private interview location. Participants completed a short demographic questionnaire following written informed consent. Interviews followed a standardized guide and were conducted in the participant's language of choice, audio recorded, transcribed, and translated to English (when conducted in another language). Guides focused on product experiences and adherence challenges and included disclosure of the participant's TFV plasma PK information [19]. Guides also included discussion topics on alternative products and preferences, with probes to explore reasons for selecting various products. Product preference selections by each participant were captured on a case report form (CRF) by the interviewer.

**Figure 1:**
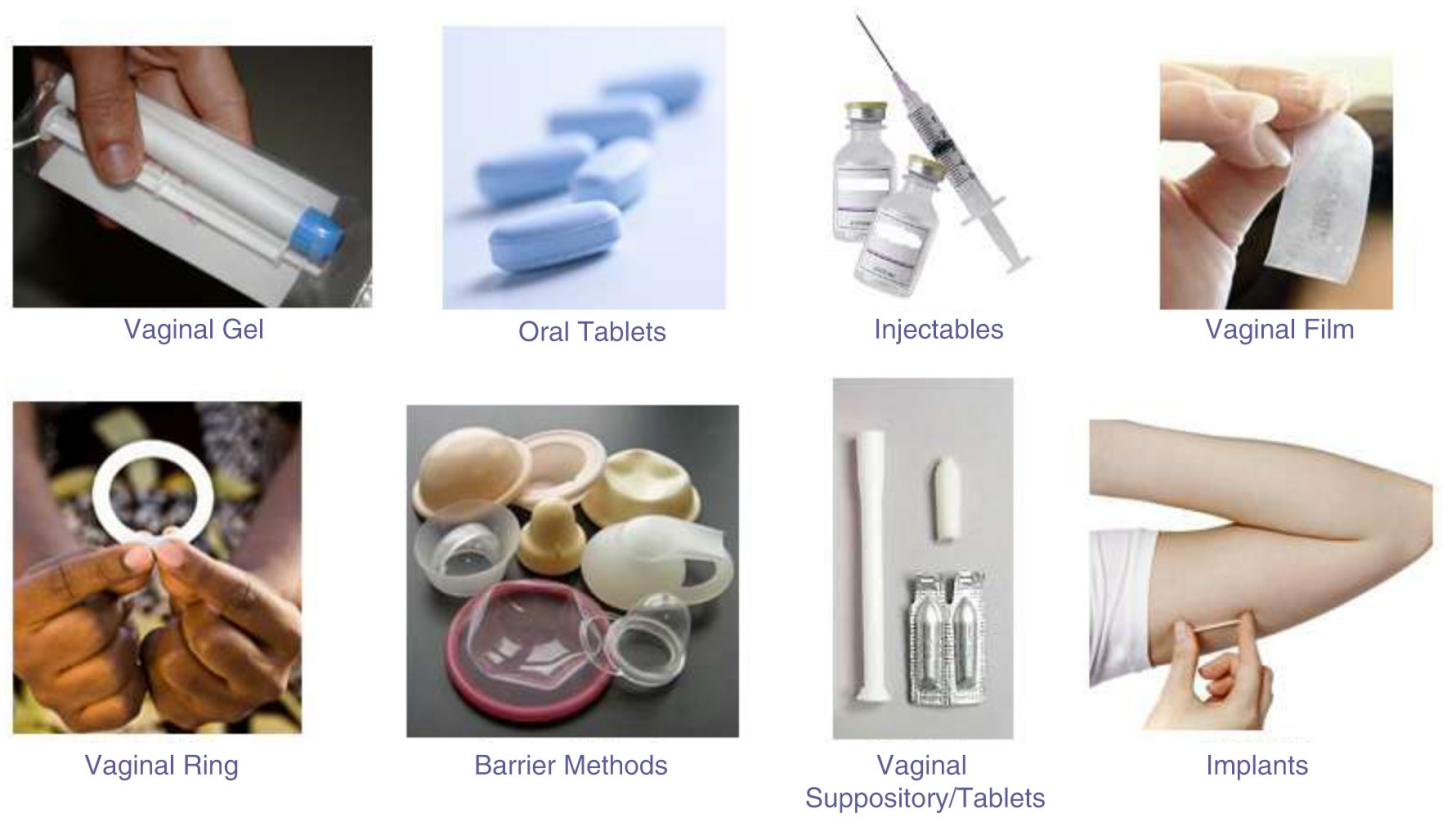
MTN-003D stage 2 HIV prevention potential product formulation discussion card. Women were shown the discussion tool and asked “What type of product would you take, if any, for HIV prevention?”

Women were shown photographs and given descriptions of eight potential PrEP product formulations – including the oral tablet and vaginal gel tested in VOICE – and asked to select what type of product formulation, if any, they would be interested to take and explain their choice(s) (Figure 1). Women could select as many products as they wanted. The eight products were cervical barrier methods, implants, injectables, oral tablets, vaginal gel, vaginal film, vaginal ring, and vaginal suppository. The key attributes of the potential product formulations were described as follows: vaginal gel – vaginally administered using an applicator, inserted daily or pericoitally; oral tablets – orally administered, used daily; injectables – injected using a needle, administered once every two to three months; vaginal film – vaginally administered using fingers, inserted daily or pericoitally; vaginal ring – vaginally administered using fingers, replaced once a month or less frequently (once every three months); barrier methods (cervical barriers) – vaginally inserted using fingers, used coitally but may be used continuously if removed, cleaned, and reinserted daily; vaginal suppository/tablets – vaginally administered using finger or an applicator, inserted daily or pericoitally; implants – flexible plastic rods placed under the skin of the upper arm, for contraception implants are inserted every three to five years.

The study protocol was approved by the Institutional Review Boards at RTI International and at each of the study sites, and was overseen by the regulatory infrastructure of the U.S. National Institutes of Health and the Microbicide Trials Network.

### Data analysis

The English versions of transcripts from IDIs were coded in Nvivo (version 10, Burlington, MA) by a four-person multisite analysis team using a codebook adapted from the VOICE-C study [20]. Throughout the coding process, high inter-coder reliability (≥80%) was established and maintained as previously described [19,20]. Coded data were combined into reports by thematic area (e.g. PREFERENCE, PRODUCT ATTRIBUTES, and REGIMEN), then summarized into memos. Memos were further analysed to reveal patterns related to product preference.

Descriptive statistics were used to present quantitative data. When relevant, data were categorized by preferred product categories. All quantitative data analyses were conducted using SAS (version 9.4, Cary, NC). Seven of the eight potential PrEP products presented to women on the potential product formulation discussion card were combined into four preferred product categories based on exploratory factor analysis (EFA) and latent class analysis (LCA). EFA utilizes the data to group products that are similar, while LCA uses the data to group women who prefer the same methods. Cervical barrier methods were excluded from these analyses because they were selected by very few women and complicated the number of preferred product categories when they were included in EFA and LCA. The four categories most closely match the three-factor solution using EFA with barrier methods removed and were consistent with the LCA findings.

Demographic and behavioural variables were selected a priori to examine correlation with each of the four preferred product formulation categories. These variables were drawn from the VOICE baseline questionnaire (VOICE study product assignment, HIV serostatus, PK group, baseline contraception, study disclosure to primary sex partner, worried about becoming HIV positive in the next year, engaged in sex work, and variables combined into a risk score) and VOICE-D demographic questionnaires (VOICE study site, age, marital status, number of lifetime sex partners, parity, educational attainment, earning her own income, male partner provides financial support, and variables combined into a socio-economic status (SES) indicator variable). Several of the continuous variables were dichotomized or split into categorical variables using the same cut-offs as the previous analyses with VOICE data (e.g. age ≤25, parity <2, PK group low/inconsistent; detailed in [8]). An SES indicator variable was created using principal component analysis (PCA) of 10 demographic assets from the VOICE-D CRF [21]; a tri-level categorical variable (lowest 40%, middle 40%, and highest 20%) was created based on the first eigenvalue and the SAS-generated PRIN1 score. The risk-scoring tool to predict HIV acquisition was developed using data from the VOICE trial (detailed in [22]). Given the small sample size and exploratory nature of these analyses, all statistical tests were univariate and used Fisher's exact tests with a significance level <0.05.

## Results

Of the 68 VOICE-D female participants who completed IDIs (22 South Africa, 22 Uganda, 24 Zimbabwe), median age was 28 (range 21–41), and nearly half (49%) were married or living with a partner (Table 1).

**Table 1:** Characteristics of VOICE-D in-depth interview participants

At time of VOICE-D interview	*N*=68	(%)
Country (VOICE study site)		
South Africa (Durban)	22	32
Zimbabwe (Chitungwiza)	24	35
Uganda (Kampala)	22	32
Age ≤25	18	26
Married or living with a partner	33	49
Number of lifetime sex partners		
1	14	21
2 to 5	38	56
6+	16	24
Parity <2	23	34
Completed secondary school or more	31	46
Earns her own income	53	78
Male partner provides financial support	58	85
Socio-economic status scale		
Lowest 40%	27	40
Middle	23	34
Highest 20%	18	26
**During VOICE trial**	***N*=68**	**(%)**

VOICE study product assignment		
Oral tablet	32	47
Vaginal gel	36	53
HIV negative	55	81
PK group		
Low/inconsistent	48	71
High	20	29
Baseline contraception		
IUD or implants	6	9
Oral contraceptives	12	18
Injectables	50	74
Disclosed study participation to primary sex partner	50	74
Worried about becoming HIV positive in the next year (baseline)	47	69
Engaged in sex work (baseline through follow-up)	13	19
High-risk score^a^ (7–12 vs. low 0–6)	26	38

a The risk score was developed using data from the VOICE trial (detailed in [22]) using multivariable modelling included age, married/living with a partner, partner provides financial or material support, partner has other partners, alcohol use, detection of a curable sexually transmitted infection, and herpes simplex virus-2 serostatus.

**Figure 2:**
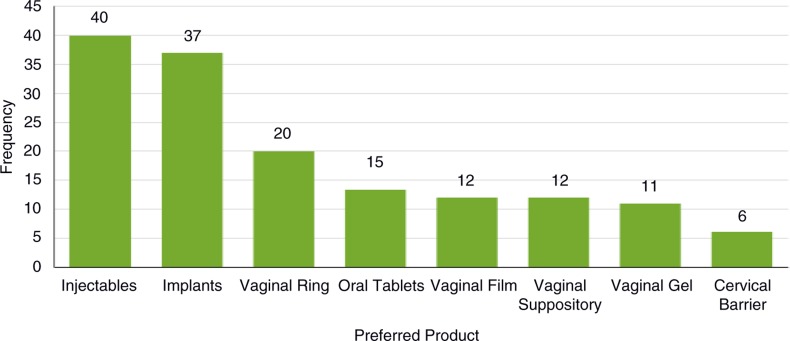
Preferred HIV prevention product formulations. Women were presented with a card showing images of eight potential HIV prevention product formulations and selected which product formulations, if any, they would prefer to use.

When prompted to select which potential product formulations, if any, they would prefer to use for HIV prevention, women selected a variety of product formulations, with injectable and implantable formulations selected most frequently (Figure 2). IDI participants chose between zero and six product formulations (mean=2.3, median=2).

**Figure 3:**
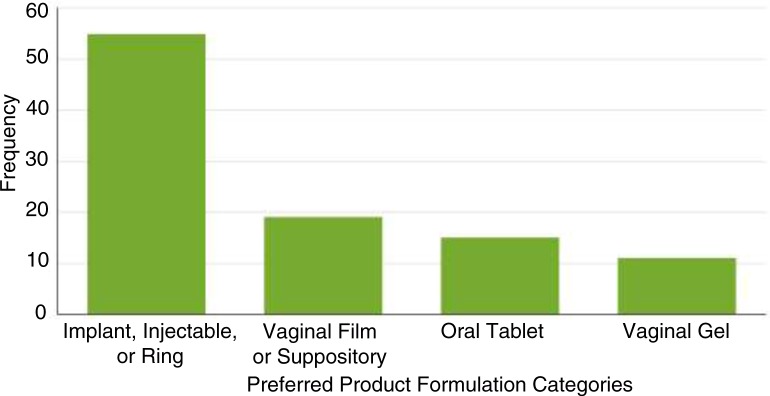
Preferred product formulation categories. Women were categorized into preferred product formulation categories by expressing a preference for any of the included methods.

The four preferred product formulation categories created based on characteristics of the formulations and the results of the EFA and supported by the LCA were: 1) oral tablets; 2) vaginal gel; 3) long-acting formulations (injectable, implant, or vaginal ring); and 4) novel on-demand products (vaginal film or suppository). A majority of women (55 of 68, 81%) expressed a preference for product formulations included in category 3, long-acting formulations (Figure 3). IDI participants were categorized into between zero and three preferred product formulation categories (mean=1.5, median=1.0).

Characteristics significantly associated with each preferred product formulation category differed (Table 2). Over half (54%, seven of 13) of women who seroconverted during VOICE selected oral tablets as a preferred HIV prevention product formulation, while only 15% of HIV-negative women were interested in oral tablets (*p*<0.01). During IDIs, participants who seroconverted were more likely to mention that tablets were easy to swallow and less likely to mention difficulties swallowing tablets. Overall, one quarter of women interviewed mentioned concerns about taking tablets when they were not sick, taking tablets that contain ARVs, or being perceived as HIV positive because of tablet use. As explained by one participant, “People start rumours about you being HIV positive, if you take pills every day” (Age 36, Zimbabwe). These concerns were not mentioned as frequently by women who had seroconverted.

**Table 2:** Demographic and behavioural correlates of preference for the four product formulation categories

	Oral tablet	Vaginal gel	Implant, injectable, or ring	Vaginal film or suppository
Overall preferred product frequency (*n*=68)	15 (22%)	11 (16%)	55 (81%)	19 (28%)
**At time of VOICE-D interview**				
Country			***	*
South Africa (*n*=22)	5 (23%)	1 (5%)	**21 (95%)**	**11 (50%)**
Zimbabwe (*n*=24)	6 (25%)	4 (17%)	**22 (92%)**	**5 (21%)**
Uganda (*n*=22)	4 (18%)	6 (27%)	**12 (55%)**	**3 (14%)**
Age	*	*		
≤25 (*n*=18)	**8 (44%)**	**0 (0%)**	17 (94%)	7 (39%)
26+ (*n*=50)	**7 (14%)**	**11 (22%)**	38 (76%)	12 (24%)
Married or living with primary sex partner				**
Yes (*n*=33)	4 (12%)	6 (18%)	28 (85%)	**4 (12%)**
No (*n*=35)	11 (31%)	5 (14%)	27 (77%)	**15 (43%)**
Parity				*
≤1 (*n*=23)	8 (35%)	1 (4%)	21 (91%)	**11 (48%)**
2+ (*n*=45)	7 (16%)	10 (22%)	34 (76%)	**8 (18%)**
Education			*	*
Did not complete secondary school (*n*=37)	5 (14%)	7 (19%)	**26 (70%)**	**6 (16%)**
Completed secondary school (*n*=31)	10 (32%)	4 (13%)	**29 (94%)**	**13 (42%)**
Socio-economic status scale				***
Lowest (*n*=27)	5 (19%)	7 (26%)	20 (74%)	**3 (11%)**
Middle (*n*=23)	8 (35%)	3 (13%)	20 (87%)	**3 (13%)**
Highest (*n*=18)	2 (11%)	1 (6%)	15 (83%)	**13 (72%)**
**During VOICE trial**				
VOICE study product assignment		***		
Oral tablet (*n*=32)	8 (25%)	**0 (0%)**	27 (84%)	9 (28%)
Vaginal gel (*n*=36)	7 (19%)	**11 (31%)**	28 (78%)	10 (28%)
HIV serostatus	**			
HIV negative (*n*=55)	**8 (15%)**	10 (18%)	44 (80%)	14 (25%)
HIV positive (*n*=13)	**7 (54%)**	1 (8%)	11 (85%)	5 (38%)

* *p*<0.05

** *p*<0.01

*** *p*<0.001, using Fisher's exact *p* value.

Women could be categorized into more than one product formulation category. Only characteristics significantly associated with each preferred product formulation category are presented.

Women aged 25 years or younger were significantly more likely to select the oral tablet (44% vs. 14%, *p*<0.05). While women of all ages expressed discomfort with swallowing oral tablets, a much higher proportion of the young women (over half) mentioned that tablets are easy to use: “Tablets are easy; you just swallow it with water” (Age 22, South Africa). Younger women also liked the portability of tablets, which can be taken on the go: “You can drink a pill even in a taxi or anywhere. You can take it with you anywhere” (Age 22, Zimbabwe). Older women were significantly more likely to select the vaginal gel (22% vs. 0%, *p*<0.05), and preference for the vaginal gel was also significantly associated with vaginal gel VOICE study product assignment (31% vs. 0%, *p*<0.001). The younger women were more likely to express concerns with the gel leaking and causing itching or other minor side effects, while women over age 25 with experience with the gel were far more likely to mention a positive impact on sexual enjoyment for themselves or their partner. One woman explained how the gel helped address vaginal dryness attributed to her family planning injection: “I would find sex easier than it was before I started using the gel. The family planning I was on had made me dry and I had lost the sex urge but when I would insert the gel, I would find having sex easier” (Age 29, Uganda). Tablet assignment during VOICE was not associated with any product formulation preference category.

While there was broad interest in implants, injectables, and vaginal rings, fewer women at the Ugandan study site (55%) selected one of these methods while most women at the South African (95%) and Zimbabwean (92%) study sites selected at least one of these methods (*p*<0.001). Women who did not complete secondary school were also significantly less likely to select these long-acting methods as preferred product formulations (70% vs. 94%, *p*<0.05). Interest in the vaginal film or vaginal suppository was significantly associated with being from South Africa, not living with a primary sex partner, lower parity (≤1 birth), having completed secondary school or higher, and high SES.

Characteristics not significantly associated with any of the preferred product formulation categories include number of lifetime sex partners, earning her own income, male partner providing financial support, PK group (low/inconsistent vs. high), baseline contraception (IUD or implant, oral contraceptives, injectables), disclosed study participation to primary sex partner, worried about becoming HIV positive in the next year (baseline), engaged in sex work (baseline through follow-up), and risk score (high 7 to 12 vs. low 0 to 6).

Participants’ explanations for their preferred product formulation selections included several key topics. Women were very concerned about their ability to adhere to daily regimens and often expressed a preference for product formulations that are long acting or on demand to fit their needs. When one woman who selected the ring was asked to explain her choice, she simply stated: “Because it lasts a long time of course” (Age 28, South Africa). Another woman indicated that a variety of product formulations would be acceptable if they lasted for a month: “It's okay if it's for a month. The pill as well if it is for a month, the same goes for the injection if it was for a month” (Age 32, South Africa). Participants also explained that clinic-administered product formulations would improve adherence and reduce unintended misuse of products (e.g. not properly discharging the full dose of vaginal gel from the applicator). Product formulations administered at the clinic by providers were also attractive because women would not have to think about storing or hiding their products at home, where they would be at risk of discovery by partners, family members, friends, or children.

Ease of use was a frequently raised topic, but how it was defined differed between women, for example, some women felt that oral tablets were easy and convenient while others found the tablets to be nearly impossible to swallow and preferred the gel. Perceptions about ease of use often seemed to be related to familiarity with product formulations, with women who were aware of different forms of contraceptive products (e.g. oral contraceptive tablets, Jadelle implant, and contraceptive injections) often expressing interest in similar potential PrEP formulations. Familiarity with the tablets and gel also grew during the VOICE study, with some women citing initial concerns with their ability to swallow the tablets or insert the gel vaginally, but then becoming comfortable with the process over the course of the study.

Some women's preference was shaped by partner concerns, including product formulation attributes which might improve or detract from sexual pleasure, product formulations which could be used without partner knowledge, or product formulations that could be used on demand by women with intermittent periods of sexual activity or in the context of specific partners. Women had differing opinions about the best route of administration, with some women preferring systemically delivered product formulations because they felt fully protected with drug circulating in their body and others interested in a product formulation that is vaginally delivered for more localized protection. There were, however, more women who indicated that they were opposed to inserting products into their vagina (due to discomfort with the process, concerns about impacts on fertility, or concerns about genital or uterine cancer) compared to those who preferred a vaginally delivered product. Illustrative quotes from the IDIs are presented in Table 3, by key topics.

**Table 3:** Quotes illustrating key topics in explanations for preferred product formulation selections

Key topic	Illustrative quotes
Dosing regimen	*The issue of using a product daily is a problem since one may forget or when it is time to take the tablets, they be in another place and you may have forgotten them at home. (Age 36, Zimbabwe)* *The problem was that we had to use it every day [in VOICE]. It would have been better if we only had to use it when we were going to do “something” [have sex]. (Age 29, South Africa)*
Clinic administered	*What's happening is that if you have to use the products on your own there is the chance that you might not use them so I think the products which are inserted at the clinic are better. (Age 30, Zimbabwe)* *Because it is a doctor who inserts [the implant] into my arm. For the others that need insertion, you have to take them home and insert them yourself. Sometimes, you might not insert the product properly. That is what happened with the gel. (Age 26, Uganda)*
Ease of use	*I think gel is difficult to use because there are so many things involved. Whereas, with the tablets, there are no difficulties at all. It's just a matter of taking the tablets. (Age 22, Zimbabwe)* *Most people liked applying the gel. The problem was the pills […] When I tried to drink the first one, I couldn't drink it. (Age 26, South Africa)*.
Partner concerns	*I think it's easy [discussing implant or injection], no stress or hassle about anything, no one can see it or know that it's there. You are just free and protected all the time. And also if others don't want to tell their partners, they don't have to […] he won't know unless he investigates if he gets suspicious. (Age 24, Zimbabwe)* *They said that if you insert the ring correctly, you do not feel anything, except for a relative of mine who is in the study [ASPIRE]. Her husband asked her what he was feeling in the vagina. I suppose she had not inserted it properly. (Age 34, Zimbabwe)*
Route of administration	*I am scared of things that get inserted into the vagina but it is okay for others. (Age 24, South Africa)* *Because I will just take them [oral tablets] knowing that they will dissolve and circulate in the body unlike these ones. (Age 26, Uganda)*

Quotes provided may fit under multiple key topic areas, but were selected to help illustrate a particular topic.

## Discussion

Women participating in the VOICE-D study expressed interest in a variety of potential HIV PrEP product formulations, with a majority of participants preferring long-acting methods. EFA and LCA supported grouping product formulations into four preferred product formulation categories. These include one category for each of the VOICE study products, one category of long-acting product formulations (implant, injectable, and ring), and one category of novel on-demand product formulations (vaginal film and vaginal suppository). These desired product formulations align well with the recommendation from the Scientific Agenda Working Group of the Initiative for Multipurpose Prevention Technologies pipeline evaluation exercise, which led to the recommendation of developing a suite of product types to accommodate the diverse needs and preference of women including sustained release devices/vaginal rings, long-acting injectable products, and on-demand formulations (gels, films, tablets, etc.) [15]. The top two preferred products in this analysis align well with the results of a study conducted by Ipsos Healthcare assessing the potential for MPTs in Uganda, South Africa, and Nigeria, which found that injectables and implants had high resonance with women while vaginal films and vaginal rings were less popular [18].

In the VOICE-D study, the vaginal ring was viewed favourably and was the third most popular product, falling into the same category as implants and injectables, with the key explanations of preference including the fact that they are long acting and can be administered at the clinic. Based on drug detection levels, evidence of use of the vaginal ring was higher in the ASPIRE trial [10] than that of tablets or gel in the VOICE trial [8]. One contributing factor may be the long-acting formulation and the simplified use regimen where the ring is inserted and replaced at the monthly clinic visit. In qualitative data in the VOICE-D study, the concept of having a product that you can “insert and forget” was prominent when related to the ring – this applies to both the dosing schedule, and the fact that the product can be used without others knowing. In the IPM 011 study, participants reported that the ring was not noticeable to others, or even to most sexual partners [23], and in the MTN-013 trial, women reported that the ring felt “normal” during sex [24].

Explanations provided by women about their product formulation selection support existing literature on the topic, which shows that some acceptability and preference issues are specific to the product's efficacy, safety, ease of use, dosage form, use requirements, and male partners [12]. The International Partnership for Microbicides (IPM) has assessed a variety of vaginal product formulations – including rings, films, gels, tablets, and soft gel capsules – and has conducted three acceptability studies [14,25,26]. In the IPM 001 study in Tanzania and South Africa, the vaginal ring was selected as a lead candidate because it was easier to use, not coitally associated, and required low frequency of administration [25]. In the Duet study in Zimbabwe, a comparison of precoital and pericoital gel and cervical barrier use found reasons for preference included lubrication, convenience, discreetness, and being prepared for unplanned sex [16]. Factors influencing the acceptability of tablets and vaginal gel among Ugandan and South African women in the MTN-001 trial included product attributes (e.g. discreetness of pills and lubricating properties of gel), familiarity with products, discreteness, partner support, and effects on sexual health [17]. Familiarity with products has also been found to influence acceptability of a vaginal ring, with participants in the IPM 001 study in Tanzania and South Africa initially expressing concern about the ring then growing to like it [25]. Acceptability of oral TDF also grew during a trial among high-risk Ghanaian women, who found that taking the pill got easier over time [27].

Preference for each preferred product formulation category was associated with different participant characteristics. These findings are consistent with research about different PrEP formulations which has found that use-regimen preference and product attribute preferences varies among women due to personal and social circumstances [12,14,16,17]. Age was differentially associated with product formulation preference, with a significantly larger proportion of younger women selecting the oral tablet and a significantly larger proportion of older women selecting the gel. This may shed light on findings from the FACTS 001 trial where young South African women (aged 18–30) only used the gel with sex about half of the time [9].

Women in this study were presented with their individual PK data as a tool to discuss nonadherence (detailed in [28]). While the full impact of how presenting individual PK adherence data affects product formulation selections is unknown, we hypothesized that by discussing PK levels and adherence challenges, women were likely to provide a more honest account of the hypothetical or actual products they would prefer. Indeed, in VOICE-D women changed the way that they reported adherence challenges after being presented with their PK results [29], and we believe that this change in narrative is indicative of a shift away from socially desirable responses and towards their true experiences. The presentation of individual drug data to participants has become a common practice in the field of HIV PrEP research, with published results from FEM-PrEP [30], IPrEx-OLE [31], as well as the ASPIRE and MTN-017 trials.

Women who seroconverted during the trial provide a unique perspective and direct experience as the at-risk population in need of prevention methods. Their experience with the VOICE study products and their preferences about alternate product formulations may be impacted by their seroconversion, due to current experiences with oral ARVs used to treat infection. Additionally, many participants did not prefer the tablets because they are associated with HIV infection. Reduced concern about the misattribution of seropositivity may influence the acceptability of the oral tablet among HIV positive women in this study.

There are some important limitations to this research. The different number of product formulations in each category may make direct comparison between categories difficult. Because some categories have more products, the overall preference percentage may be increased when compared to a category with only one product. Indeed, the product category with the highest preference percentage (implant, injectable, and ring) also has the most product formulations. However, based on the frequency of selection of the individual product formulations, we are not concerned that the preference for the product formulation category is being biased simply due to the inclusion of more options.

Additionally, since none of these products were on the market for HIV prevention, women's choices are hypothetical as far as HIV prevention is concerned and may not translate to future HIV prevention product use, acceptability, or adherence. Actual use experience with different products may provide more accurate information about what women like and do not like about a product formulation, and what they are willing to use. In previous work with cervical cups and vaginal gels, a majority of women changed their regimen preference from baseline following actual use of the two different regimens, indicating that hypothetical preference may not be a good predictor of actual user preference [16]. In this analysis, baseline contraceptive use was not associated with product formulation preference.

It is also important to note that acceptability may not correlate with use: a product with high acceptability may not be used consistently, and products with known effectiveness may be used even if only moderately acceptable [32–35]. That said, when users are free to choose between multiple viable options, acceptability is assumed to be a key factor driving adherence [34]. The results of this exploratory analysis with a relatively small number of women who were just presented with their product use PK data in a large clinical trial, add to a growing field of acceptability and preference research, but may not be generalizable to a broader population.

## Conclusions

Research shows that women at risk of HIV infection need a range of options for HIV prevention product formulations and that these options should include different use requirements and dosage forms. In order to obtain high coverage, a broad range of HIV prevention product formulations will need to be developed that address diverse needs, as modelled by the contraceptive field. While there was interest in a variety of potential HIV PrEP product formulations, a majority of VOICE-D participants preferred long-acting methods. Analyses of the correlates of product preference along with research on various product delivery forms can inform messaging and market segmentation as well as the amount of resources to invest in different products that target populations are most interested in using. The development of PrEP products is an expensive process, with limited resources for the development, evaluation, and delivery of these products. Understanding the attributes of products women prefer will improve the likelihood of developing products that meet the diverse needs and wants of women at risk of HIV infection.
